# Habitat suitability and connectivity modeling predict genetic population structure and priority control areas for invasive nutria (*Myocastor coypus*) in a temperate river basin

**DOI:** 10.1371/journal.pone.0279082

**Published:** 2022-12-16

**Authors:** Wanmo Kang, GoWoon Kim, Yongsu Park

**Affiliations:** 1 Department of Forest Environment and Systems, College of Science and Technology, Kookmin University, Seoul, Republic of Korea; 2 Center for Asian Urban Societies, Asia Center, Seoul National University, Seoul, Republic of Korea; 3 Research Center for Endangered Species, National Institute of Ecology, Gyeongsangbuk-do, Yeongyang-gun, Republic of Korea; University of New England, UNITED STATES

## Abstract

The nutria (*Myocastor coypus*), also known as the coypu, is a semi-aquatic, invasive rodent native to South America that causes damage to natural riverine and wetland habitats in many parts of the world, including South Korea. Understanding habitat use, connectivity, and gene flow of nutria populations is critical for the sound management of local and regional ecosystems. Here, we assessed habitat suitability and connectivity in relation to the genetic structure of nutria populations in the Nakdong River Basin of South Korea. A total of 321 nutria occurrence sites and seven environmental variables were used to perform ensemble habitat suitability modeling using five species distribution models (SDMs), including boosted regression trees, maximum entropy model, random forest, generalized linear model, and multivariate adaptive regression splines. Using graph and circuit theory approaches, we assessed the population gene flow and current flow betweenness centrality (CFBC) of suitable habitats derived from the ensemble SDM. All SDMs performed well with a range of test AUC values from 0.962 to 0.970 (mean = 0.966) with true skill statistic values over 0.8. The minimum temperature of the coldest month, mean temperature of the warmest quarter, precipitation of the driest quarter, and distance from water bodies were important predictors in nutria habitat modeling. Nutria population gene flow was significantly correlated with the least-cost path distance on a cost resistance surface based on ensemble habitat suitability modeling and roads (Mantel’s *r* = 0.60, *p* < 0.05). Finally, the CFBC positively correlated with the genetic diversity of nutria populations was used to identify priority control areas. Habitat suitability and connectivity modeling not only revealed environmental conditions and areas that support the survival and spread of nutrias, but also improved our understanding of the animals’ genetic population structure, thereby indicating priority areas to target for eradication.

## Introduction

Invasive alien species (IAS) are one of the largest threats to native biodiversity, along with habitat loss and climate change [1–3]. IAS compete with other native species for limited resources, cause disease and extinction of indigenous flora and fauna, and lead to degradation and displacement of native habitats [4–6]. This can induce significant ecosystem disruption and socio-economic damage by altering ecosystem services or impacting infrastructure [7, 8]. Moreover, the management of IAS, either through control or eradication, usually requires considerable labor and financial resources [9].

Understanding the factors driving invasion success, distribution, spread pattern, and impacts is critical for the effective and efficient management of IAS [10]. Although preventing new IAS from arriving is the most cost-effective solution, the introduction of alien species and wildlife trading are continuously increasing, facilitating the spread of IAS [11, 12]. Once an IAS is established and has spread, identifying current and potential habitats and their connectivity becomes an essential task to effectively control their impact in ecosystems [13]. Because the spread of IAS is constantly increasing worldwide, despite global efforts to limit biological invasions [14], prioritizing high-risk IAS based on their potential ecological and socio-economic impacts is crucial.

The nutria (*Myocastor coypus*), also called the coypu, is a large, semi-aquatic rodent native to temperate and subtropical South America. The species was initially introduced to Africa, Asia, Europe, and North America for fur and meat farming. Due to declining demand for nutria products, nutrias have been released and flourished in natural riverine and wetland habitats in Korea and other parts of the world. The nutria has generalist feeding habits, mostly fond of aquatic plants and roots, thus damaging native vegetation and crops, resulting in changes in soil structure, and turning valuable wetlands into open water [15, 16]. Nutria burrowing and foraging behaviors further aggravate wetland and biodiversity loss by accelerating soil erosion and competing with native species, such as the muskrat in North America [17–19].

Because the nutria is recognized as a pervasive ecological threat, its establishment and spread has been identified as one of the top priority environmental issues in many places [20–22]. Accordingly, the nutria has also been listed among the top 100 of the world’s worst IAS [23]. However, the detailed distribution pattern and, by extension, the landscape genetics of the nutria remain unclear, which may provide vital clues for effectively detecting and controlling its spread. Some studies have related the occurrence of nutrias with environmental factors such as climate and topographic conditions to predict habitat suitability [24–26] or have investigated the distributional shifts of nutria populations [20, 26, 27]. Other studies have characterized the structure and genetic diversity of nutria populations for management purposes [28, 29]. However, no comprehensive study has yet examined the gene flow and habitat connectivity of nutria populations as well as their habitat distribution, which can serve as a key functional element in the development of spatial management plans and strategies for nutria control. Connectivity and gene flow, in turn, shape the genetic structure pattern of a species, which is of profound evolutionary and ecological importance [30, 31]. Overall, the habitat suitability and gene flow network for nutrias are poorly understood, which hinders the implementation of IAS management to effectively isolate and eradicate nutria populations.

**Fig 1:**
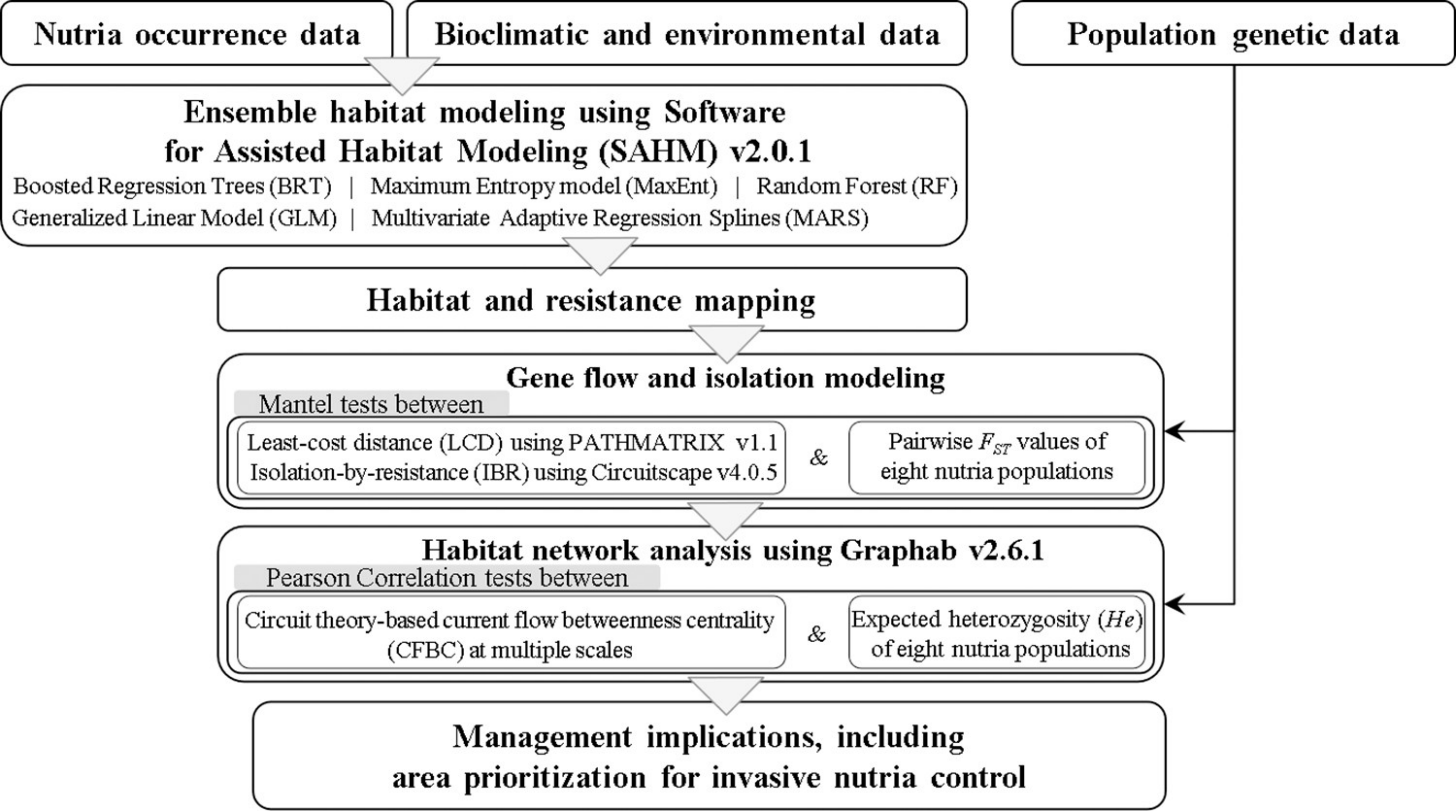
The overall research process of this study.

To overcome these limitations, we developed nutria habitat suitability and connectivity models based on the occurrence of nutrias, bioclimatic and environmental data, and population genetic data. Focusing on this invasive species, we demonstrated how models combining habitat and genetic data can be used to predict species distribution and spatial population structure. We specifically aimed to assess habitat suitability, gene flow connectivity, and the habitat network of nutria populations in the Nakdong River Basin of South Korea. Using ensemble species distribution models along with graph and circuit theory approaches, we identified important environmental factors influencing nutria distribution, assessed habitat suitability and gene flow connectivity, examined the functional habitat network, and prioritized key areas to control the spread of this IAS in South Korea. The overall research process and outcomes of this study are shown in Fig 1.

## Methods

### Study area

**Fig 2:**
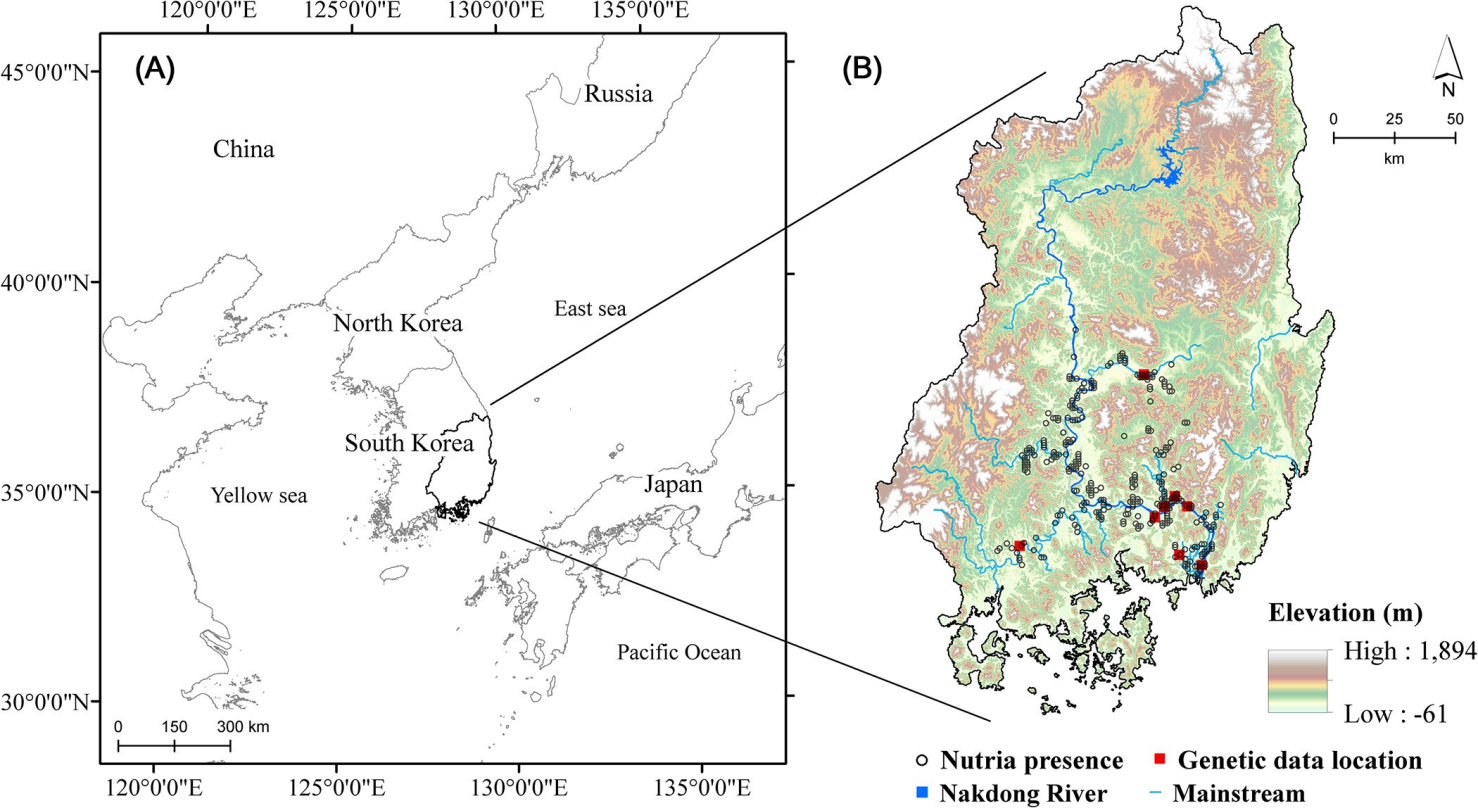
Geographical location and elevation of the Nakdong River Basin in Korea (base map data source: https://gadm.org/). (A) The location of the Korean Peninsula in Northeast Asia. (B) Digital elevation model (DEM) of the Nakdong River Basin and its rivers and mainstreams, with occurrence sites (n = 321) and genetic data locations (n = 8) of nutrias indicated as open black circles and closed red squares, respectively. The shuttle radar topography mission (SRTM) DEM with 30-m resolution was provided by the USGS Earth Explorer platform (https://earthexplorer.usgs.gov/). The genetic data location coordinates were obtained from the study by Kim et al. [28]. River and stream data were derived from the OpenStreetMap database (http://www.openstreetmap.org).

The study area was comprised of the Nakdong River Basin and surrounding islands in Korea, lying between 34.5–37.3°N, and 127.4–129.6°E, WGS 84 (Fig 2A). The Nakdong River is the longest river in South Korea (approximately 510 km) (Fig 2B). The study area was approximately 31,669 km^2^ with an average elevation of approximately 266 m above mean sea level. Forests cover 68.4% of the Nakdong River Basin; upland fields and paddy fields cover 7.2% and 10.4% of the basin, respectively; with the remaining areas covered by grasslands, bare lands, and urban areas (0.1–6.9%) [32]. The study area had a typical temperate climate with four distinct seasons affected by East Asian monsoons.

### Nutria occurrence sites

Between 2016 and 2020, field surveys of the study area were conducted at a walking speed of 0.5–2.0 km/h, more than twice every quarter, i.e., each season, by 10–15 wildlife experts of the Nakdong River Basin Environmental Office (Nakdong River BEO, Ministry of Environment of Korea). The locations of nutria occurrences based on direct and indirect observations, such as dens, droppings, and footprints, were noted during the field surveys. All sites were recorded using GPS coordinates. To minimize location overlaps and maintain compatibility with spatial data resolution, all nutria occurrence sites were separated by a minimum distance of 1 km. A total of 321 nutria occurrence sites were obtained (Fig 2B).

### Species distribution modeling

Seven bioclimatic and environmental variables were selected based on their relevance to nutria habitat distribution according to previous studies [25–27, 33] (Table 1). Among them, three bioclimatic variables with a spatial resolution of 30 arc-seconds (1 km) were included: the minimum temperature of the coldest month, the mean temperature of the warmest quarter, and the precipitation of the driest quarter, averaged from 1970–2000 [34]. As a ground vegetation biomass variable, the median normalized difference vegetation index (NDVI) at 30 m spatial resolution was generated using all Landsat-8 operational land imager (OLI) data from 2016–2020 via the Google Earth Engine (GEE) platform [35]. Distance from water bodies was included as a predictor of foraging activity [36]. Two anthropogenic factors were also used as human impact proxies, including distance to roads and human population density. The OpenStreetMap (OSM) database (as of November 2017) provided road vector data, including motorways, trunks, and primary, secondary, and tertiary roads [37]. As a population density variable, population grid data of 1 km resolution was obtained from the geospatial information platform (http://map.ngii.go.kr) for October 2018. To maintain consistency with other data, the NDVI variable was resampled to a 1 km resolution using bilinear interpolation. To examine multicollinearity, Pearson’s cross-correlations were calculated between all variable pairs based on the 321 nutria occurrence sites and 10,000 randomly generated points across the study area. No variables were highly correlated (|*r*| < 0.7 for all pairs) [38].

**Table 1:** Bioclimatic and environmental variables used in ensemble species distribution modeling.

Variable	Range	Spatial resolution	Temporal coverage	Data source
**Minimum temperature of coldest month**	-13.6~2.3 (°C)	1 km	1970–2000	WorldClim database version 2 (http://www.worldclim.com/version2)
**Mean temperature of warmest quarter**	16.6~24.9 (°C)	1 km	1970–2000	WorldClim database version 2 (http://www.worldclim.com/version2)
**Precipitation of driest quarter**	61.3~156.8 (mm)	1 km	1970–2000	WorldClim database version 2 (http://www.worldclim.com/version2)
**Median normalized difference vegetation index (NDVI)** ^**a**^	-0.2~0.8 (unitless)	30 m	2016–2020	Landsat-8 operational land imager (OLI) imagery
**Distance from water bodies**	0.0~24.6 (km)	1 km	2016	Digital map (1:25,000)
**Distance to roads**	0~10.3 (km)	1 km	2017	OpenStreetMap database (http://www.openstreetmap.org)
**Human population density**	0~27,341 (people/km^2^)	1 km	2018	Geospatial information platform (http://map.ngii.go.kr)

^a^Variable resampled to 1 km resolution

An ensemble of predictions of five species distribution model (SDM) algorithms was employed to identify the major environmental factors influencing nutria distribution and potential suitable habitats, using Software for Assisted Habitat Modeling (SAHM) v.2.0.1 for VisTrails v.2.2.3 [39]. The SAHM software provides a workflow interface that enables easily to fit SDMs, to calculate model performance metrics, to explore variable importance and response curves, and to perform model selection. The five SDM models included boosted regression trees (BRT), maximum entropy model (MaxEnt), random forest (RF), generalized linear model (GLM), and multivariate adaptive regression splines (MARS). A presence-only method using background data was employed to fit the five models [40].

The 321 nutria occurrence sites were combined with environmental factors in the SDMs for habitat suitability predictions. A total of 10,000 background data points were randomly generated, excluding nutria occurrence sites within the study area. For BRT, alpha = 1, bag fraction = 0.75, number of folds = 3, and default settings for the other values were used. For MaxEnt, the default settings in the SAHM package were used. For RF, the out-of-bag prediction error was minimized using the tuneRF function. For GLM, a bidirectional stepwise procedure based on the Akaike information criterion (AIC) was used, considering all interactions and squared terms. For MARS, Mars degree (Friedman’s μ) = 1 and generalized cross-validation penalty per knot = 2.0 were used [41].

All SDMs were evaluated based on a 70–30% train-test data split using the area under the receiver operating characteristic (ROC) curve (AUC) and true skill statistic (TSS) [42]. The AUC (a threshold-independent evaluation metric) ranges from 0 to 1, with higher values indicating better model performance [43]. The TSS (a threshold-dependent evaluation metric) ranges from -1 to 1, with higher values indicating better model performance [42]. The TSS was calculated using the threshold that maximized the sum of sensitivity and specificity in the ROC curve [44, 45]. Ten-fold cross-validation was applied to the training dataset for model selection. Only models with an average test AUC value > 0.75 and TSS value > 0.4 were selected for further analyses [46].

The variable importance for each model was examined using the change in AUC values with and without the independent variable, but using all other variables [47]. The increase in AUC (ΔAUC) was calculated when each variable was permuted for each model. Variable importance was then ranked by the mean ΔAUC of the five models and model response curves were generated for the four most important variables with ΔAUC ≥ 0.05.

To obtain a robust ensemble model estimate, model predictions were consolidated using the committee averaging method [48] in which model predictions for occurrence probability were translated into binary form (1 = suitable, 0 = unsuitable) using the threshold that maximized the sum of sensitivity and specificity [44, 45]. The binary prediction models were then averaged to create a final ensemble model of habitat suitability [49].

### Gene flow and isolation modeling

The fixation index (*F*_*ST*_) is a widely used genetic distance measure for genetic differentiation [50] which occurs when gene flow between populations is restricted. Pairwise *F*_*ST*_ values of eight nutria populations in the Nakdong River Basin of South Korea, comprised of 93 individuals each typed at 12 microsatellite loci, were obtained from the study by Kim et al. [28] (Fig 2B). These authors reported that variation in *F*_*ST*_ values was not explained by pairwise Euclidean distance (i.e., isolation-by-distance, IBD). Therefore, to estimate the presence of genetic isolation by other advanced distance measures, the *F*_*ST*_ genetic distance was evaluated against the least-cost distance and the resistance distance among population pairs [51, 52]. The least-cost distance (LCD) is the accumulated cost distance of the optimal pathway connecting population pairs. The resistance distance (i.e., isolation-by-resistance, IBR) considers all possible pathways connecting population pairs based on circuit theory [52]. To measure the two distance metrics, a 50 m resolution resistance surface was generated by assigning exponentially decreasing cost values (ranging from 1–100) to the habitat suitability values from the ensemble model as follows: a cost value of 100 assigned to 0 (least suitable), 40 to 0.2, 15 to 0.4, 5 to 0.6, 2 to 0.8, and 1 to 1 (most suitable). In addition, a cost of 10,000 was assigned for major roads since these seem to be one of the most disruptive barriers to nutria migration [53]. The effect of roads on gene flow was also examined by either including or not including the road cost.

LCDs were calculated using PATHMATRIX software [54]. Resistance distances were measured using Circuitscape v4.0.5 software [55]. The significance of correlations between *F*_*ST*_ and distance measures were evaluated using Mantel tests [56], and were computed using the vegan package in R with 10,000 permutations [57].

### Habitat network analysis and area prioritization

A habitat network map was created to identify priority areas for nutria control. Graph and circuit theory approaches were used to map the habitat network, providing effective methods to evaluate multiple aspects of habitat connectivity and priority areas [58, 59]. The habitat network (i.e., graph) was defined as a set of suitable habitat patches (nodes) and connections between them (links). In the final ensemble SDM, nutria occurrence probabilities above 0.5 were considered suitable (at least three of the five SDMs predicted as suitable). We rasterized the suitable pixel center points of the ensemble prediction map at a 50 m resolution, and defined them as habitat patches.

As a precautionary principle, the degree of connectivity should be overestimated rather than underestimated when connectivity is viewed negatively [60]. Thus, least-cost modeling to find the optimal connectivity solution is considered a better way to measure connectivity for invasive species. For this reason, links in the network, representing the functional relationships between habitat patches in terms of potential migration or movement of nutrias, were defined by a cumulative cost distance using the 50 m resolution cost surface, which used also for the gene flow and isolation modeling. The cumulative least-cost distances between habitats were within the maximum cost distance of 250,000 for computational efficiency. The habitat network was assumed to be a thresholded complete graph that directly connected all pairs of nodes with less than a specified threshold cost distance. Nutria dispersal and home range distances, also known as the population’s spreading rate, are known in Euclidean form, ranging from hundreds of meters to tens of kilometers [61]. Moreover, the home range size and dispersal distance of nutrias can be largely dependent on resource availability and environmental conditions [61, 62]. Thus, multiple cost distances from 5,000–25,000 with intervals of 5,000 were employed to threshold the habitat network.

The connectivity metric (current flow betweenness centrality (CFBC) [63]) was calculated to determine priority areas for nutria control. CFBC applies a circuit theory algorithm [64]. Links on a regular lattice (e.g., raster grid) correspond to resistors, each of which has its own resistance. In this study, current (i.e., flow of charge), which can be measured through a resistor, represented the movement probability of a random walker through least-cost paths. CFBC is determined only by the resistance and number of links. The metric requires only one parameter (e.g., patch capacity) that influences the distribution of CFBC along the nodes and links of the graph. In this study, the capacity acted as the amperage of the source patches. According to the patch definition, the amperage was set to 1 for all patches (β = 0). The CFBC estimates of each habitat network thresholded at the different cost distances were measured at the patch level. To determine the optimal cost distance scale in relation to the genetic structure of nutria populations, Pearson correlations were computed between expected heterozygosity (*He*, also called gene diversity) and CFBC measured at each threshold cost distance. Due to lack of normality, the CFBC estimates were log-transformed for Pearson correlation analysis. *He* values of eight nutria populations were obtained from the study by Kim et al. [28]. Each nutria population’s CFBC corresponded to that of the nearest habitat patch to each genetic data locality. High-priority control areas were identified as those within the top 5% of CFBC derived at the most significant scale relating nutria genetic diversity with habitat connectivity. Habitat network analyses were conducted using Graphab v2.6.1 software [65]. ArcGIS v10.4.1 software was used for mapping [66] and IBM SPSS Statistics 25 [67] to perform correlation tests.

## Results

All five SDMs performed well in predicting nutria occurrence, with test AUC values ranging from 0.962–0.970 with a mean of 0.966. MaxEnt (AUC = 0.970) had the highest predictive capability, followed by RF (AUC = 0.968), BRT (AUC = 0.965), MARS (AUC = 0.963), and GLM (AUC = 0.962). The TSS values of all models ranged from 0.825–0.888. When assessing model performance using a ten-fold cross-validation on the training dataset, all five SDMs had good predictive power, with a mean test AUC ≥ 0.954 and TSS ≥ 0.442. Thus, the predictions of all five SDMs were combined into an ensemble SDM using the committee averaging method.

**Fig 3:**
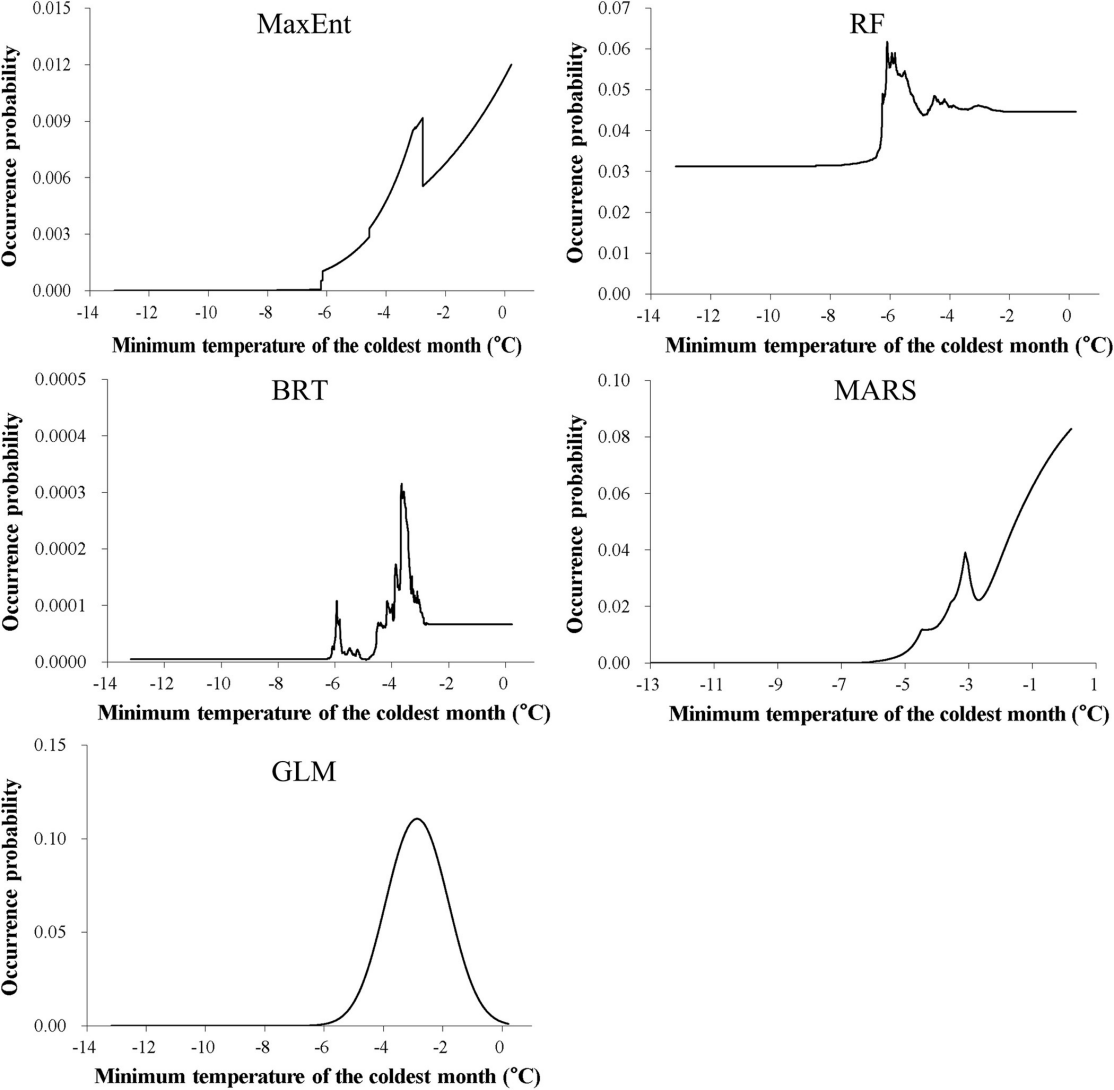
Response curves for the most important predictor, the minimum temperature of the coldest month for the five species distribution models for nutrias (producing ΔAUC values ≥ 0. 05). MaxEnt = Maximum Entropy; RF = Random Forest; BRT = Boosted Regression Trees; MARS = Multivariate Adaptive Regression Spline; GLM = Generalized Linear Model.

**Fig 4:**
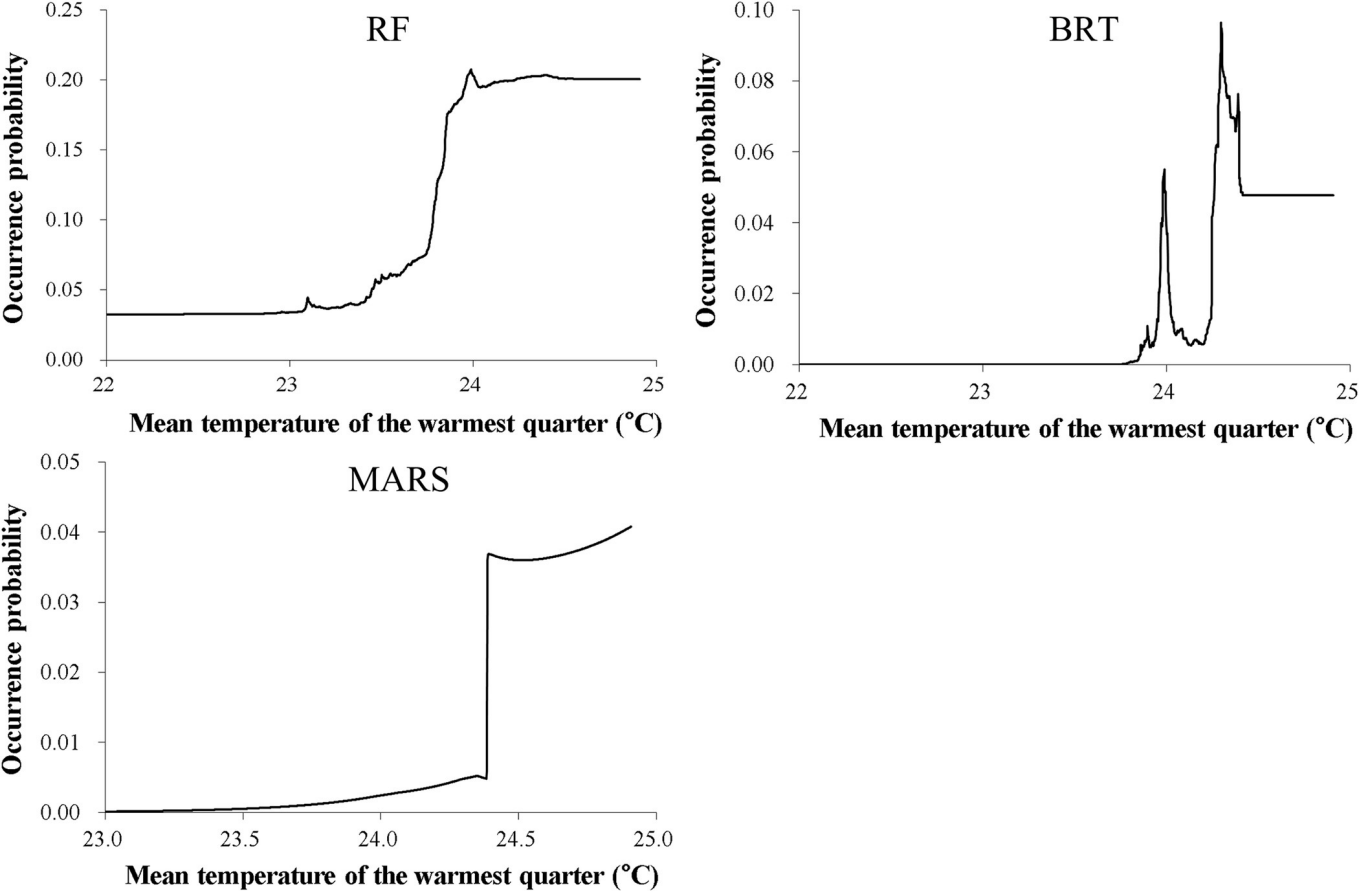
Response curves for the second important predictor, the mean temperature of the warmest quarter for three of the five species distribution models for nutrias (producing ΔAUC values ≥ 0. 05). RF = Random Forest; BRT = Boosted Regression Trees; MARS = Multivariate Adaptive Regression Spline.

**Fig 5:**
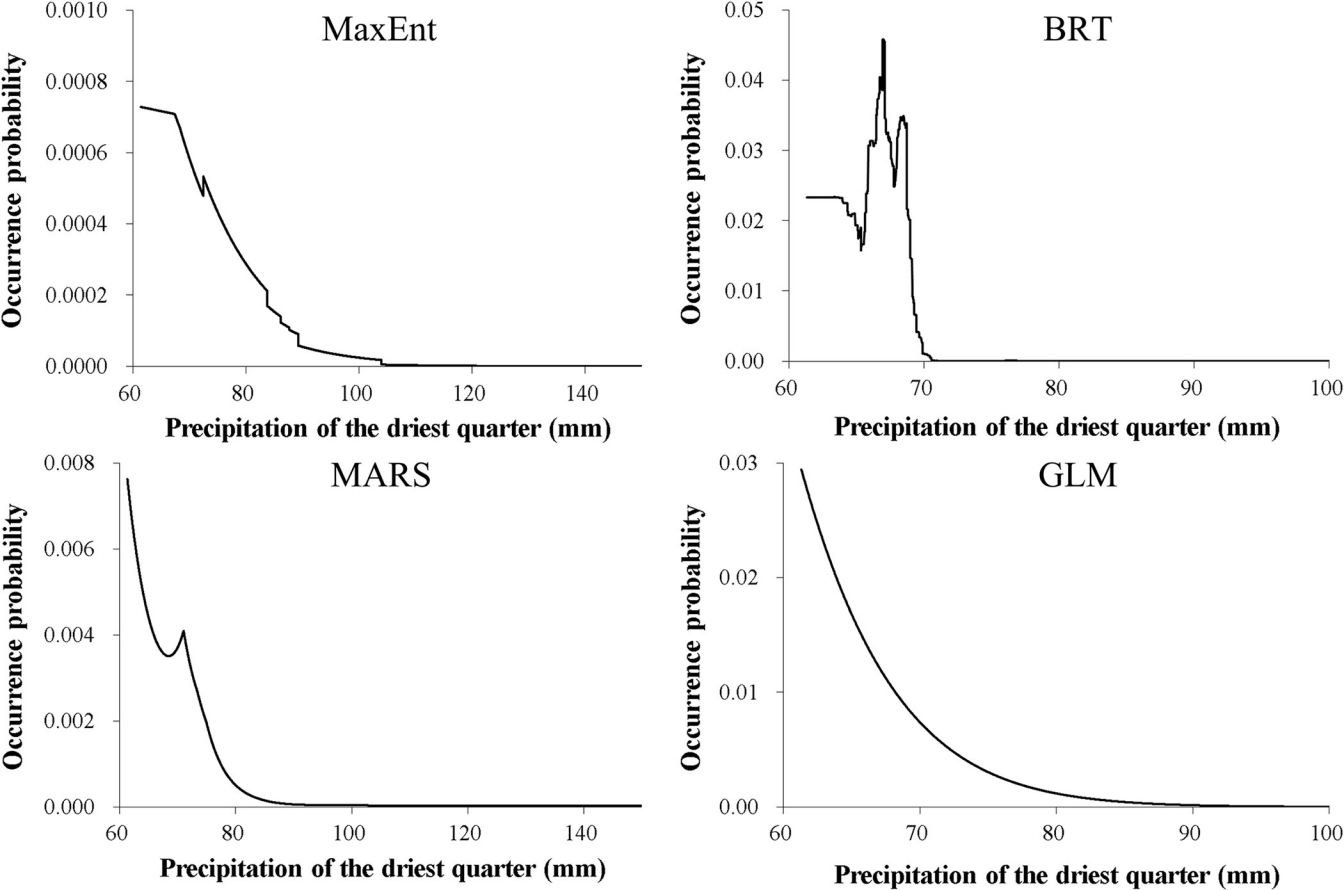
Response curves for the third important predictor, the precipitation of the driest quarter for four of the five species distribution models for nutrias (producing ΔAUC values ≥ 0. 05). MaxEnt = Maximum Entropy; RF = Random Forest; MARS = Multivariate Adaptive Regression Spline; GLM = Generalized Linear Model.

**Fig 6:**
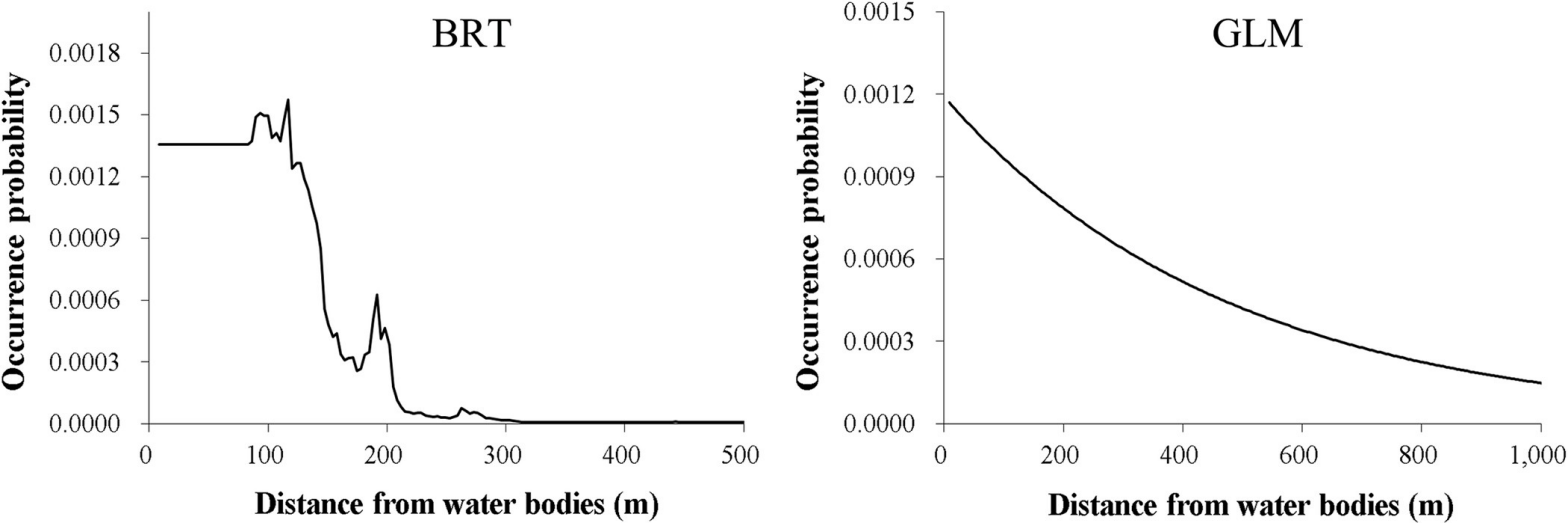
Response curves for the fourth important predictor, distance from water bodies for two of the five species distribution models for nutrias (producing ΔAUC values ≥ 0. 05). BRT = Boosted Regression Trees; GLM = Generalized Linear Model.

Among the seven predictors, the minimum temperature of the coldest month was the most important variable (Table 2). For most SDMs, this predictor generally had a positive influence on nutria occurrence at minimum temperatures higher than approximately -6°C, while the probability of occurrence was nearly 0 at minimum temperatures lower than approximately -6°C (Fig 3). As the second most important variable (Table 2), the mean temperature of the warmest quarter generally had a positive effect on nutria occurrence at mean temperatures higher than approximately 23.5°C (Fig 4). The precipitation of the driest quarter and distance from water bodies were identified as other important variables (Table 2) negatively correlated with nutria occurrence (Figs 5 and 6). Median NDVI, distance to roads, and population density were not important predictors of nutria occurrence (i.e., ΔAUC values < 0.01).

**Table 2:** Relative variable importance for each species distribution model using an increase in AUC values (ΔAUC).

Variable	MaxEnt	RF	GLM	BRT	MARS	Mean ΔAUC
**Minimum temperature of the coldest month**	0.140	0.105	0.232	0.110	0.299	0.177
**Mean temperature of warmest quarter**	0.048	0.060	0.045	0.055	0.247	0.091
**Precipitation of driest quarter**	0.081	0.036	0.122	0.073	0.082	0.079
**Distance from water bodies**	0.034	0.048	0.054	0.053	0.027	0.043
**Median normalized difference vegetation index (NDVI)**	0.009	0.020	0.008	0.000	0.000	0.007
**Distance to roads**	0.007	0.016	0.000	0.000	0.000	0.005
**Population density**	0.002	0.009	0.007	0.000	0.000	0.004

MaxEnt = Maximum Entropy; RF = Random Forest; GLM = Generalized Linear Model; BRT = Boosted Regression Trees; MARS = Multivariate Adaptive Regression Spline.

**Fig 7:**
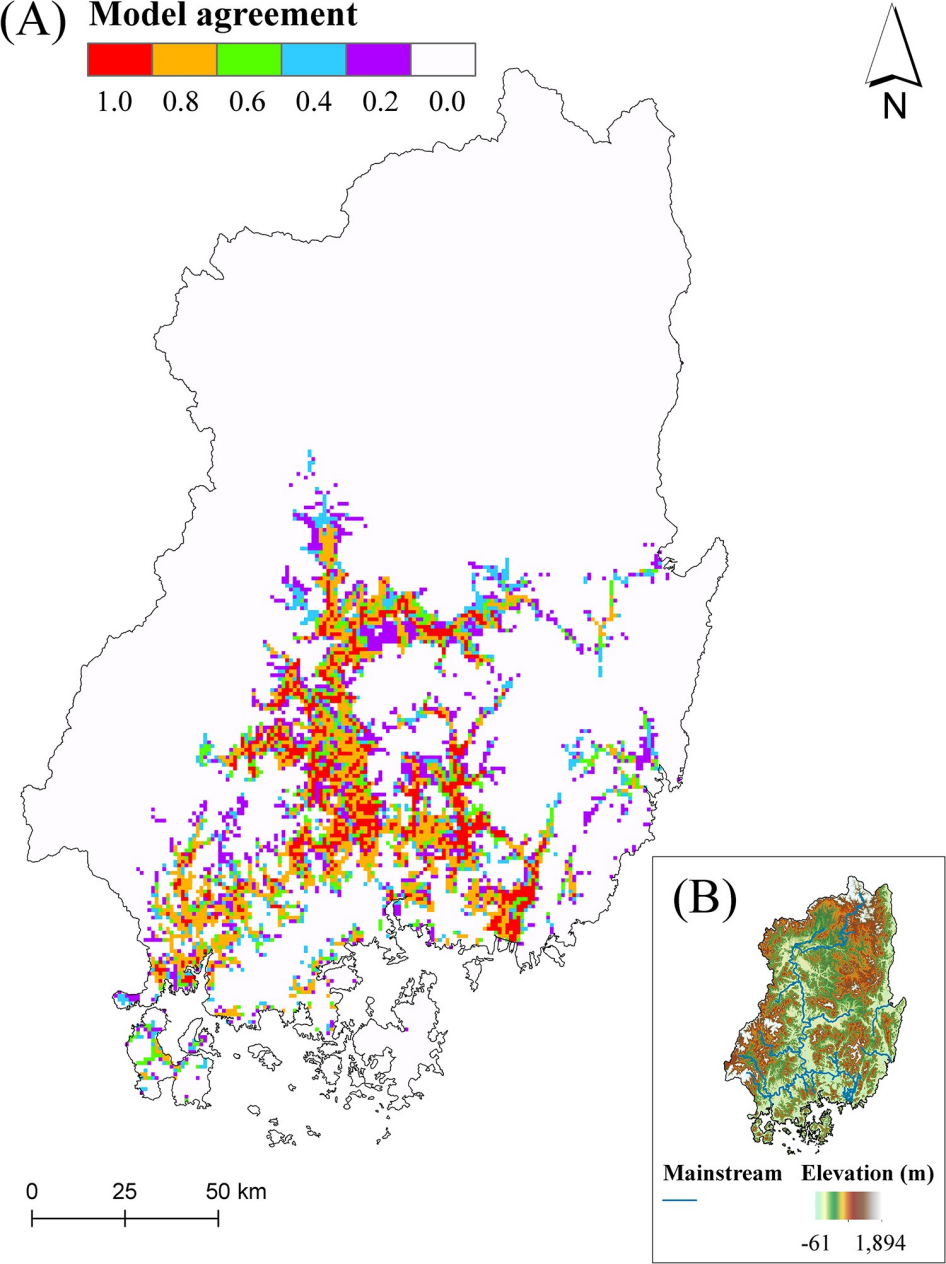
Ensemble map for the probability of nutria occurrence across the Nakdong River Basin in Korea (A) using binary predictions of five species distribution models (base map data source: https://gadm.org/). Different colors indicate the level of habitat suitability consensus among the predictions of five species distribution models. For example, red indicates that all five models predicted the area as suitable, while white indicates that all five models predicted the area as unsuitable. (B) Inlet map showing a digital elevation model (DEM) of the Nakdong River Basin and its mainstreams (base map data source: https://gadm.org/). The shuttle radar topography mission (SRTM) DEM with 30-m resolution was provided by the USGS Earth Explorer platform (https://earthexplorer.usgs.gov/). Stream data was derived from the OpenStreetMap database (http://www.openstreetmap.org).

Fig 7 presents the ensemble map derived from the five SDMs for nutria occurrence in the Nakdong River Basin, Korea. Suitable nutria habitats predicted by all and ≥ 3 models covered 2.84 and 9.73% of the total basin area, respectively. Conversely, unsuitable habitats predicted by all and ≥ 3 models covered 83.85 and 90.27% of the total area, respectively. The most suitable nutria habitats (i.e., core habitats) were in the middle and downstream lowland areas of the Nakdong River Basin (Fig 7).

**Fig 8:**
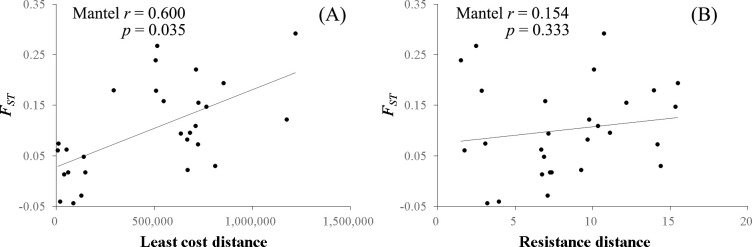
Mantel correlations between *F*_*ST*_ and (A) least-cost distance and (B) resistance distance calculated for eight nutria populations in the Nakdong River Basin.

**Fig 9:**
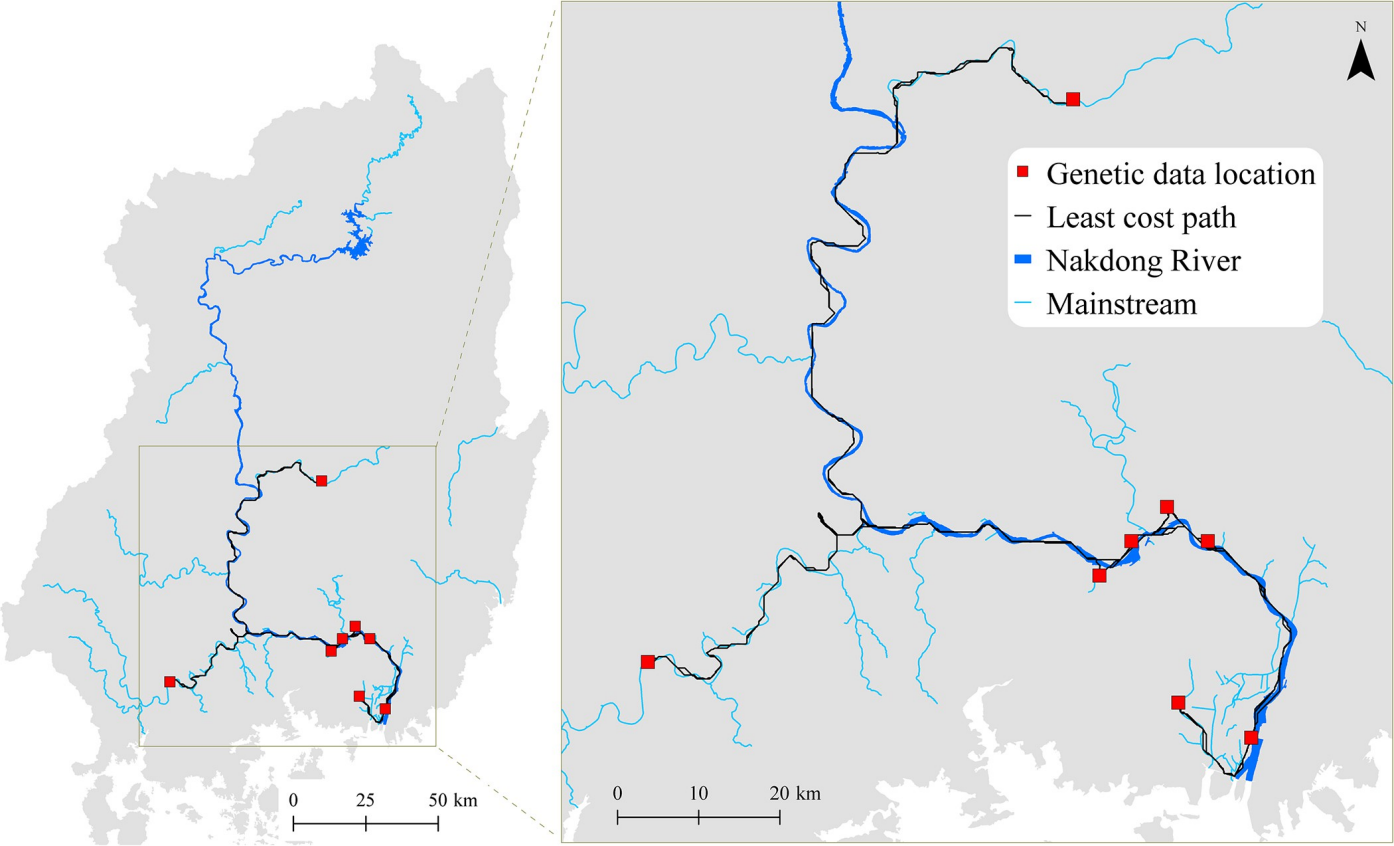
Least-cost paths connecting pairs of eight nutria populations in the Nakdong River Basin (base map data source: https://gadm.org/). The middle and downstream area was enlarged. The genetic data location coordinates were obtained from the study by Kim et al. [28]. River and stream data were derived from the OpenStreetMap database (http://www.openstreetmap.org).

**Fig 10:**
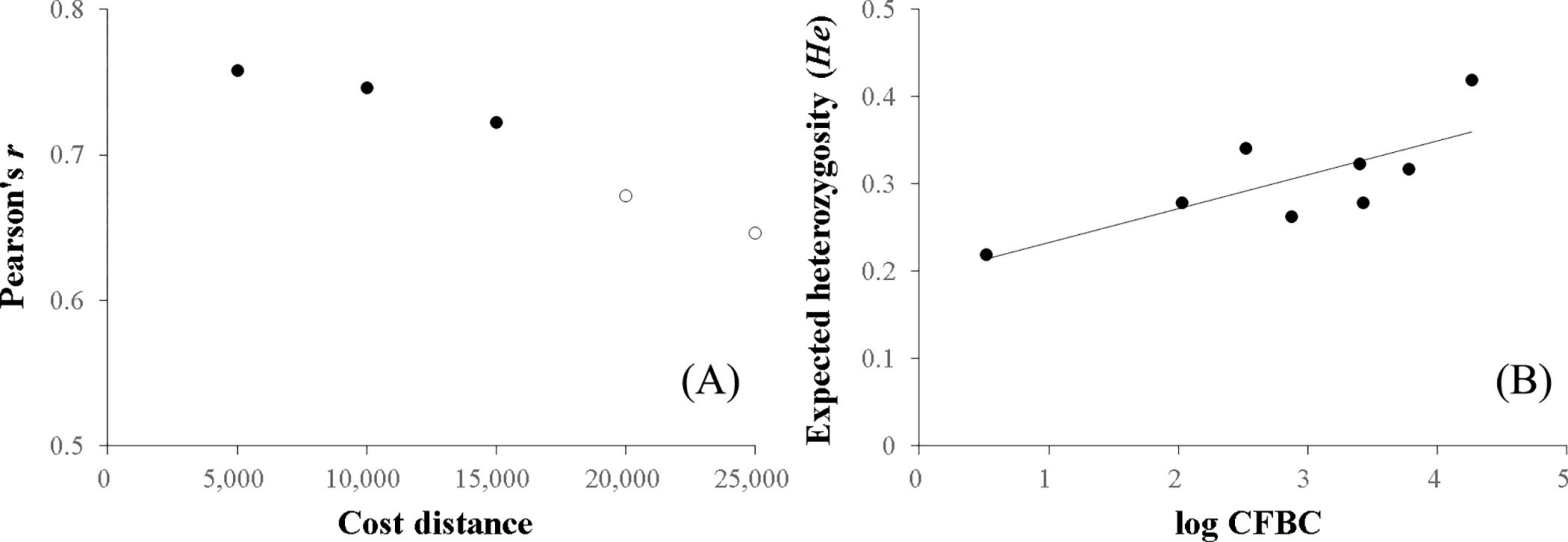
Relationship between the genetic diversity of nutria populations and their habitat connectivity. (A) Pearson’s *r* coefficients between the expected heterozygosity (*He*) of eight nutria populations and their habitat current flow betweenness centrality (CFBC) measured at each cost distance threshold of 5,000 to 25,000 with intervals of 5,000 in the Nakdong River Basin. Closed circles indicate significant correlations (*p* < 0.05). The strongest correlation was observed at a cost distance of 5,000. (B) Scatter plot and fit line between *He* and CFBC measured at the cost distance threshold of 5,000.

**Fig 11:**
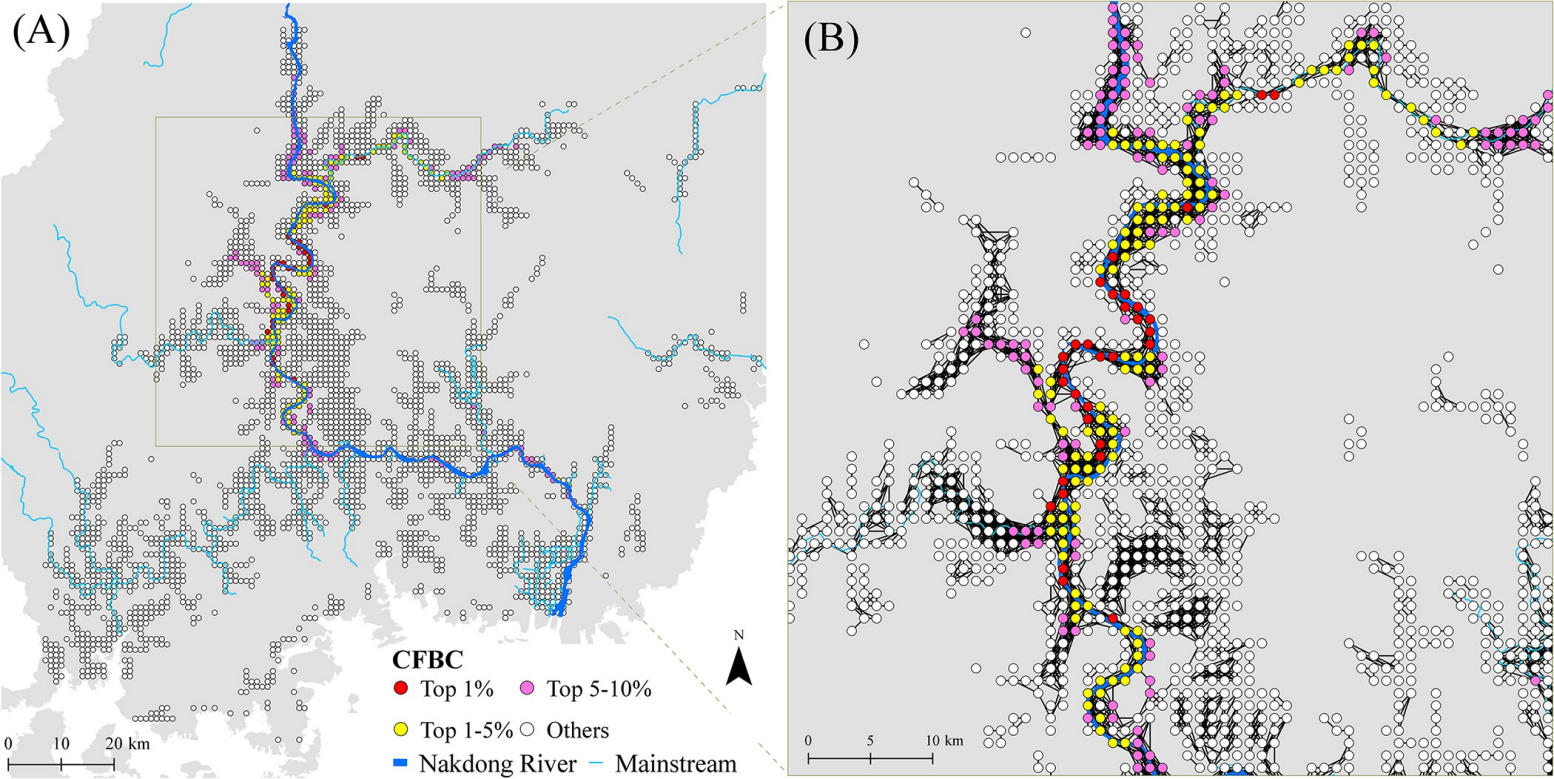
Connectivity level (current flow betweenness centrality, CFBC) of habitat nodes for genetic connectivity of nutria populations (base map data source: https://gadm.org/). (A) CFBC of habitat nodes measured at a cost distance threshold of 5,000 within the middle and downstream regions of the Nakdong River Basin. Circles represent habitat nodes. Red and yellow circles indicate priority habitat nodes of high connectivity for invasive species management. (B) Enlarged view of a key control area including high-priority habitat nodes along the Nakdong River and its major adjacent tributaries. Links connecting habitat nodes are also shown. River and stream data were derived from the OpenStreetMap database (http://www.openstreetmap.org).

The *F*_*ST*_ genetic distance was significantly correlated with LCD based on habitat suitability and road cost values (Mantel *r* = 0.600, *p* < 0.05), but not with the IBR (Mantel *r* = 0.154, *p* = 0.333) (Fig 8). Least cost pathways connecting nutria populations mainly followed the Nakdong River and its major tributaries (Fig 9). However, genetic distance was not correlated with LCD when road cost was not included (Mantel *r* = 0.110, *p* = 0.359). The *He* values of the eight nutria populations were significantly correlated (*p* < 0.05) with their habitat CFBC measured at distance thresholds of 5,000–15,000 cost units (Fig 10A). The most significant correlation between the *He* values of the eight nutria populations and CFBC (*r* = 0.758, *p* < 0.05) occurred when the distance threshold was set at 5,000 cost units (Fig 10B). Thus, the distance threshold for the network analyses was set at 5,000 cost units. The map of the CFBC pinpointed high-priority control areas, including the top 10% of habitat nodes with high connectivity along the midstream areas (Fig 11). Riparian habitats in the mainstream section of the Nakdong River and its major adjacent tributaries, approximately 100 km long, were identified as key control areas for the prevention of nutria spread and eradication.

## Discussion

This study is the first comprehensive modeling assessment to predict habitat distribution and priority control areas for invasive nutrias in a temperate river basin. The study findings indicate that habitat suitability was largely determined by the minimum temperature of the coldest month, mean temperature of the warmest quarter, precipitation of the driest quarter, and distance from water bodies. Further, the level of least-cost connectivity based on an ensemble model and road network was significantly related to the genetic differentiation of nutria populations. In addition, the genetic diversity of nutria populations was positively related to the degree of node connectivity in the species habitat network.

Importance analysis of environmental variables highlighted two temperature-related climatic factors—the minimum temperature of the coldest month and the mean temperature of the warmest quarter—as being crucial in shaping habitat suitability for the nutria. Nutria occurrence was more likely in areas of mild climate with the minimum temperature of the coldest month and the mean temperature of the warmest quarter above approximately -6 and 23.5°C, respectively. This finding concurs with that of other studies reporting temperature as a potential limiting factor of spread [20, 26] since nutrias are particularly vulnerable to cold winters [27]. Indeed, nutrias tend to thrive and propagate rapidly in areas with mild winters [62, 68]. Although cold weather has been identified as a significant factor limiting the reproduction and range expansion of nutrias [25, 69, 70], ongoing climate change may lead to range expansion of the species [26].

The precipitation of the driest quarter and distance from water bodies also played an important role in shaping the distribution of the species. The probability of nutria occurrence decreased with increasing levels of precipitation in the driest quarter. In the study area, the driest quarter broadly coincides with the cold season. Thus, the precipitation of the driest quarter can be considered a proxy for snow cover or depth, which may hinder animal movement and feeding [71]. Distance from water bodies also had a negative effect on nutria distribution. This result is consistent with the ecological characteristics of nutrias, which rely on riparian habitats. Previous studies have also indicated the importance of water resources (e.g., rivers and streams) for the distribution of this species [25, 27, 33]. Meanwhile, the NDVI variable was not important in the SDMs, even though vegetation cover provides food and shelter [33, 61, 72]. Considering the ecological characteristics of the nutria, the combined effect of vegetation cover and water resources on habitat suitability should be evaluated using methods such as the ecological niche factor analysis (ENFA) [33]. The other variables, including distance to roads and population density, also had lower importance values, as reported in other studies [26, 33].

The ensemble distribution map (Fig 7) revealed that nearly 10% of the Nakdong River Basin in Korea can be considered suitable nutria habitats, predicted by over half of the SDMs. The map also illustrated that nutrias prefer lowland riverine areas compared to mountainous areas [33]. However, the predicted suitable habitats were confined to midstream and downstream riverine areas of the basin with relatively mild winters. Due to continuous global warming, temperatures in the basin are expected to rise, most notably in upstream regions in winter [73]. Although no occurrence was recorded in the upstream regions in this study or in other studies [20, 27], upstream areas adjacent to suitable nutria habitats are at risk of invasion. Therefore, these areas warrant further attention and monitoring.

Population structure in landscape genetics is commonly assessed by IBD, examining the relationship between Euclidean inter-population geographic distance and genetic distance [74]. However, the current study applied LCD and IBR models as more sophisticated landscape genetic approaches to evaluate the effects of spatial habitat suitability and roads on genetic connectivity. Although Kim et al. [28] reported that variation in *F*_*ST*_ values for eight nutria populations was not explained by IBD, a significant positive correlation was determined in the current study between genetic and LCD on a resistance surface based on an ensemble habitat suitability model and roads (Fig 8A). This result indicates that gene flow of nutria populations is highly constrained by the spatial configuration of habitat suitability and roads. Further, least cost path modeling suggested that potential gene flow routes mainly follow the Nakdong River and its major tributaries. In addition, genetic distance was not significantly correlated with LCD when major roads were not considered movement barriers, whereas distance to roads had a low importance in SDMs. Overall, these suggest that nutria can occur close to roads, but roads are significant barriers to nutria migration. Thus, suitable habitat areas with less road coverage should be prioritized for the control and prevention of nutria spread.

Circuit theory-based IBR often predicts population genetic structures better than IBD and LCD [75]. Circuit theory, which is based on random-walk theory, assumes that animals have no knowledge of the landscape (i.e., the relative resistance of landscape features) beyond their immediate surroundings [76]. In contrast, least-cost modeling assumes that animals have knowledge of the landscape and associated costs in order to choose the lowest cost path [51]. Interestingly, unlike LCD, IBR was not supported in this study (Mantel *r* = 0.154, *p* = 0.333). Nutrias spread or disperse mainly along rivers and tributaries [27] and tend to remain in the vicinity of their natal area for their entire lives (i.e., philopatric behavior) [77]. These characteristics suggest that a cost resistance surface mainly based on habitat suitability ranging from 0 to 1 may not be appropriate for IBR to evaluate the contribution of multiple pathways to the gene flow of this species.

High habitat connectivity may increase genetic diversity through increased gene flow [78]. Accordingly, the study results demonstrated that the degree of habitat connectivity based on network flow (i.e., CFBC) was positively correlated with the genetic diversity of nutria populations. Moreover, habitat connectivity was related to the genetic diversity of the species in a scale-dependent manner. *He* values of eight nutria populations were significantly correlated (*p* < 0.05) with their habitat CFBC, measured at distance thresholds of 5,000 to 15,000 cost units (Fig 10A). This may correspond to the range of scales at which nutrias perceive habitats that are functionally connected in the middle and downstream regions of the Nakdong River Basin, which might be associated with species traits, such as movement behavior and perceptual range, as well as environmental conditions. Despite their philopatric behavior, nutrias are known to travel tens of kilometers when resources are limited [25, 79]. However, due to suitable environmental conditions for nutria survival in this region, the most significant correlation was found at a relatively short cost distance of 5,000 units (corresponding to a Euclidean distance of approximately 2.5 km).

Priority areas to control the establishment and spread of nutrias were explicitly identified using a CFBC map as the backbone of habitat connectivity in the Nakdong River Basin (Fig 11). Key control areas of high habitat connectivity included riparian habitats in the mainstream section of the Nakdong River and its major adjacent tributaries. These areas pinpointed where surveillance and control efforts, such as hunting and trapping, should be targeted to effectively and efficiently eradicate and isolate nutria populations. Surveillance and control of these areas, especially those close to high biodiversity and protected areas, are critically important for ecosystem conservation and restoration.

## Conclusions

The nutria poses a serious ecosystem threat due to its impacts on biodiversity and water resources. Targeted actions against species invasion should be based on a reliable, integrated, and spatially explicit understanding of habitat requirements and ecological processes, as well as available control options. To this end, this study combined ensemble habitat suitability modeling, landscape genetics, and habitat network analysis to create a functional habitat network map as an integrated approach for the prevention of nutria spread and its eradication. This habitat network map is expected to play a pivotal role in identifying priority control areas and guiding the planning and management of invaded and adjacent non-invaded riparian areas to reduce the distribution and spread of nutrias. This study provides a cost-effective and efficient approach to better allocate resources for continuous surveillance, monitoring, and control of invasive species at local or regional levels. Because species distribution variability may occur at relatively smaller scales, in the future, high resolution local environmental data are required to perform more accurate habitat suitability and connectivity modeling for nutria control. Further research should also be directed to better understand individual movement and space-use behavior patterns of this species across spatiotemporal scales (i.e., movement ecology) in a changing landscape and climate.

## Supporting information

S1 File The WGS84 geographic coordinates of nutria occurrence locations used to fit the five species distribution models. (ZIP)

## References

[1] Chapin FS, Zavaleta ES, Eviner VT, Naylor RL, Vitousek PM, Reynolds HL, et al. Consequences of changing biodiversity. Nature. 2000;405: 234–242. doi: 10.1038/35012241 10821284

[2] Simberloff D, Martin J-L, Genovesi P, Maris V, Wardle DA, Aronson J, et al. Impacts of biological invasions: what’s what and the way forward. Trends Ecol Evol. 2013;28: 58–66. doi: 10.1016/j.tree.2012.07.013 22889499

[3] Lockwood JL, Hoopes MF, Marchetti MP. Invasion Ecology. Malden, MA: Blackwell; 2013.

[4] Cherry TL, Shogren JF, Frykblom P, List JA. Valuing wildlife at risk from exotic invaders in Yellowstone Lake. In: Alberini A, Kahn JR, editors. Handbook on Contingent Valuation. Northampton, MA: Edward Elgar; 2009. pp. 307–323.

[5] Veblen TT, Mermoz M, Martin C, Kitzberger T. Ecological impacts of introduced animals in Nahuel Huapi National Park, Argentina. Conserv Biol. 1992;6: 71–83.

[6] Shea K, Chesson P. Community ecology theory as a framework for biological invasions. Trends Ecol Evol. 2002;17: 170–176.

[7] Bacher S, Blackburn TM, Essl F, Genovesi P, Heikkilä J, Jeschke JM, et al. Socio-economic impact classification of alien taxa (SEICAT). Methods Ecol Evol. 2018;9: 159–168.

[8] Pejchar L, Mooney HA. Invasive species, ecosystem services and human well-being. Trends in Ecology and Evolution. 2009;24: 497–504. doi: 10.1016/j.tree.2009.03.016 19577817

[9] Hoffmann BD, Broadhurst LM. The economic cost of managing invasive species in Australia. Neobiota. 2016;31: 1–18.

[10] Taylor NG. Why are invaders invasive? Development of tools to understand the success and impact of invasive species. PhD Dissertation, University of Leeds. 2016.

[11] Lockwood JL, Welbourne DJ, Romagosa CM, Cassey P, Mandrak NE, Strecker A, et al. When pets become pests: the role of the exotic pet trade in producing invasive vertebrate animals. Front Ecol Environ. 2019;17: 323–330.

[12] García-Díaz P, Ross JV, Woolnough AP, Cassey P. The illegal wildlife trade is a likely source of alien species. Conserv Lett. 2017;10: 690–698.

[13] Stewart-Koster B, Olden JD, Johnson PTJ. Integrating landscape connectivity and habitat suitability to guide offensive and defensive invasive species management. J Appl Ecol. 2015;52: 366–378.

[14] Seebens H, Blackburn TM, Dyer EE, Genovesi P, Hulme PE, Jeschke JM, et al. No saturation in the accumulation of alien species worldwide. Nat Commun. 2017;8: 14435. doi: 10.1038/ncomms14435 28198420 PMC5316856

[15] Carter J, Leonard BP. A review of the literature on the worldwide distribution, spread of, and efforts to eradicate the coypu (*Myocastor coypus*). Wildl Soc Bull. 2002;30: 162–175.

[16] Kendrot S. Restoration through eradication: protecting Chesapeake Bay marshlands from invasive nutria (*Myocastor coypus*). In: Veitch CR, Clout MN, Towns DR, editors; 2011. IUCN, Gland, Switzerland. pp. 313–319.

[17] Taylor KL, Grace JB, Marx BD. The effects of herbivory on neighbor interactions along a coastal marsh gradient. Am J Bot. 1997;84: 709–715. 21708623

[18] Shaffer GP, Day JW, Hunter RG, Lane RR, Lundgerg CJ, Wood WB, et al. System response, nutria herbivory, and vegetation recovery of a wetland receiving secondarily-treated effluent in coastal Louisiana. Ecol Model. 2015;79: 120–131.

[19] Boscareno JM. The rise and fall of the Louisiana muskrat, 1890–1960: an environmental and social history. New Orleans, Louisiana, USA. University of New Orleans. 2009.

[20] Kim Y-C, Kim A, Lim J, Kim T-S, Park S-G, Kim M, et al. Distribution and management of nutria (*Myocastor coypus*) populations in South Korea. Sustainability. 2019;11: 4169.

[21] Kruse RC. The Impact of Nutria (*Myocastor Coypus*) as an Invasive Species and Its Possible Distribution in Washington State. Evergreen State College. 2012.

[22] Sasser CE, Holm GO, Evers-Hebert E, Shaffer GP. The nutria in Louisiana: a current and historical perspective. In: Day JW, Erdman JA, editors. Mississippi Delta restoration: pathways to a sustainable future: Springer, Cham; 2018. pp. 39–60.

[23] Global Invasive Species Database (GISD). 100 of the world’s worst invasive alien species. 2004 Nov [cited 21 Oct 2021]. Available from: http://www.iucngisd.org/gisd/100_worst.php.

[24] Kim A, Kim Y-C, Lee D-H. A management plan according to the estimation of nutria (*Myocastor coypus*) distribution density and potential suitable habitat. J EIA. 2018;27: 203–214 (in Korean with English abstract).

[25] Sheffels TR. Status of Nutria (*Myocastor coypus*) Populations in the Pacific Northwest and Development of Associated Control and Management Strategies, with and Emphasis on Metropolitan Habitats. Thesis for degree of Doctor of Philosophy. Portland State University, Portland, USA. 2013.

[26] Schertler A, Rabitsch W, Moser D, Wessely J, Essl F. The potential current distribution of the coypu (*Myocastor coypus*) in Europe and climate change induced shifts in the near future. NeoBiota. 2020;58: 129–160.

[27] Hong S, Do Y, Kim JY, Kim D-K, Joo G-J. Distribution, spread and habitat preferences of nutria (*Myocastor coypus*) invading the lower Nakdong River, South Korea. Biol Invasions. 2015;17: 1485–1496.

[28] Kim IR, Choi W, Kim A, Lim J, Lee D-H, Lee JR. Genetic diversity and population structure of nutria (*Myocastor coypus)* in South Korea. Animals. 2019;9: 1164. 31861229 10.3390/ani9121164 PMC6940949

[29] Klima K, Travis S. Genetic population structure of invasive nutria (*Myocastor coypus*) in Louisiana, USA: is it sufficient for the development of eradication units? Biol Invasions. 2012;14: 1909–1918.

[30] López-Márquez V, Templado J, Buckley D, Marino I, Boscari E, Micu D, et al. Connectivity among populations of the top shell Gibbula divaricata in the Adriatic Sea. Frontiers in Genetics. 2019;10. 30906312 10.3389/fgene.2019.00177 PMC6418013

[31] Laurence S, Smith MJ, Schulte-Hostedde AI. Effects of structural connectivity on fine scale population genetic structure of muskrat, *Ondatra zibethicus*. Ecol Evol. 2013;3: 3524–3535. 24223287 10.1002/ece3.741 PMC3797496

[32] Lee J, Lee Y, Woo S, Kim W, Kim S. Evaluation of water quality interaction by dam and weir operation using SWAT in the Nakdong river Basin of South Korea. Sustainability. 2020;12: 6845.

[33] Farashi A, Najafabadi MS. A model to predict dispersion of the alien nutria, *Myocastor coypus* Molina, 1782 (Rodentia), in Northern Iran. Acta Zool Bulg. 2017;69: 65–70.

[34] Fick SE, Hijmans RJ. Worldclim 2: new 1-km spatial resolution climate surfaces for global land areas. Int J Climatol. 2017;37: 4302–4315.

[35] Gorelick N, Hancher M, Dixon M, Ilyushchenko S, Thau D, Moore R. Google Earth Engine: Planetary-scale geospatial analysis for everyone. Remote Sens Environ. 2017;202: 18–27.

[36] Borgnia M, Galante ML, Cassini MH. Diet of the Coypu (Nutria, *Myocastor coypus*) in Agro-Systems of Argentinean Pampas. J Wildl Manage. 2000;64: 354–361.

[37] OpenStreetMap contributors. OpenStreetMap South Korea. Available at https://download.geofabrik.de/asia/south-korea.html [Accessed 21 September 2017]. 2015.

[38] Dormann CF, Elith J, Bacher S, Buchmann C, Carl G, Carré G, et al. Collinearity: a review of methods to deal with it and a simulation study evaluating their performance. Ecography. 2013;36: 27–46.

[39] Morisette JT, Jarnevich CS, Holcombe TR, Talbert CB, Ignizio D, Talbert MK, et al. VisTrails SAHM: visualization and workflow management for species habitat modeling. Ecography. 2013;36: 129–135.

[40] Franklin J. Mapping species distributions: spatial inference and prediction. New York, US: Cambridge University Press; 2009.

[41] Friedman J. Multivariate adaptive regression splines. Ann Stat. 1991;19: 1–67.

[42] Allouche O, Tsoar A, Kadmon R. Assessing the accuracy of species distribution models: prevalence, kappa and the true skill statistic (TSS). J Appl Ecol. 2006;43: 1223–1232.

[43] Baldwin RA. Use of maximum entropy modeling in wildlife research. Entropy. 2009;11: 854–866.

[44] Liu C, Newell G, White M. On the selection of thresholds for predicting species occurrence with presence-only data. Ecol Evol. 2015;6: 337–348. doi: 10.1002/ece3.1878 26811797 PMC4716501

[45] Liu C, White M, Newell G. Selecting thresholds for the prediction of species occurrence with presence-only data. J Biogeogr. 2013;40: 778–789.

[46] Elith J. Quantitative methods for modeling species habitat: comparative performance and an application to Australian plants. In: Ferson S, Burgman M, editors. Quantitative methods for conservation biology. New York: Springer; 2002. pp. 39–58.

[47] Hayes MA, Piaggio AJ. Assessing the potential impacts of a changing climate on the distribution of a rabies virus vector. PLOS ONE. 2018;13: e0192887. doi: 10.1371/journal.pone.0192887 29466401 PMC5821341

[48] Araújo MB, New M. Ensemble forecasting of species distributions. Trends Ecol Evol. 2007;22: 42–47. doi: 10.1016/j.tree.2006.09.010 17011070

[49] Stohlgren TJ, Ma P, Kumar S, Rocca M, Morisette JT, Jarnevich CS, et al. Ensemble habitat mapping of invasive plant species. Risk Anal. 2010;30: 224–235. doi: 10.1111/j.1539-6924.2009.01343.x 20136746

[50] Weir BS, Cockerham CC. Estimating F-statistics for the analysis of population structure. Evolution. 1984;38: 1358–1370. doi: 10.1111/j.1558-5646.1984.tb05657.x 28563791

[51] Adriaensen F, Chardon JP, De Blust G, Swinnen E, Villalba S, Gulinck H, et al. The application of ’least-cost’ modelling as a functional landscape model. Landsc Urban Plan. 2003;64: 233–247.

[52] McRae BH. Isolation by resistance. Evolution. 2006;60: 1551–1561. 17017056

[53] Aldrovandi S, Finotti G, Milioni F, Leonardi S, Corazza C. Wildlife road mortality in a plain landscape of high conservation value (Eastern Po Valley, Northern Italy). Quaderni del Museo Civico di Storia Naturale di Ferrara. 2018;6: 99–110.

[54] Ray N. PATHMATRIX: a geographical information system tool to compute effective distances among samples. Mol Ecol Notes. 2005;5: 177–180.

[55] McRae B, Shah V, Mohapatra T. Circuitscape 4 user guide. The Nature Conservancy, Arlington. 2013 [cited 21 Oct 2021]. Available from: http://www.circuitscape.org.

[56] Mantel N. The detection of disease clustering and a generalized regression approach. Cancer Res. 1967;27: 209–220. 6018555

[57] Oksanen J, Blanchet FG, Friendly M, Kindt R, Legendre P, McGlinn D, et al. vegan: Community Ecology Package. R package version 2.5–7. 2020 [cited 21 Oct 2021]. Available from: https://CRAN.R-project.org/package=vegan.

[58] Rayfield B, Fortin MJ, Fall A. Connectivity for conservation: a framework to classify network measures. Ecology. 2011;92: 847–858. doi: 10.1890/09-2190.1 21661548

[59] Urban D, Minor E, Treml E, Schick R. Graph models of habitat mosaics. Ecol Lett. 2009;12: 260–273. doi: 10.1111/j.1461-0248.2008.01271.x 19161432

[60] Etherington TR. Least-Cost Modelling and Landscape Ecology: Concepts, Applications, and Opportunities. Curr Landsc Ecol Rep. 2016;1: 40–53.

[61] Baroch J, Hafner M, Brown TL, Mach JJ, Poché RM. Nutria (*Myocastor coypus)* in Louisiana. Wellington, Colorado: Genesis Laboratories, Inc; 2002. pp. 1–155.

[62] Gosling LM, Watt AD, Baker SJ. Continuous retrospective census of the East Anglian coypu population between 1970 and 1979. J Anim Ecol. 1981;50: 885–901.

[63] Carroll C, McRae BH, Brookes A. Use of linkage mapping and centrality analysis across habitat gradients to conserve connectivity of gray wolf populations in western North America. Conserv Biol. 2012;26: 78–87. doi: 10.1111/j.1523-1739.2011.01753.x 22010832

[64] McRae BH, Dickson BG, Keitt TH, Shah VB. Using circuit theory to model connectivity in ecology, evolution, and conservation. Ecology. 2008;89: 2712–2724. doi: 10.1890/07-1861.1 18959309

[65] Foltête J-C, Vuidel G, Savary P, Clauzel C, Sahraoui Y, Girardet X, et al. Graphab: an application for modeling and managing ecological habitat networks. Software Impacts. 2021;8: 100065.

[66] ESRI. ArcGIS, Version 10.4.1. Redlands, California, USA. 2016.

[67] IBM Corp IBM SPSS Statistics for Windows, Version 25.0. Armonk, NY: IBM Corp. 2017.

[68] Gosling LM. Climatic determinants of spring littering by feral coypus, *Myocastor coypus*. J Zool. 1981;195: 281–288.

[69] Runami I, Gunji Y, Hishinuma M, Nagano M, Takada T, Higaki S. Reproductive biology of the coypu, *Myocastor coypus* (Rodentia: Myocastoridae) in western Japan. Zoologia (Curitiba). 2013;30: 130–134.

[70] Reggiani G, Boitani L, D’Antoni S, De Stefano R. Biology and control of the coypu in the Mediterranean area. Supplementi alle Ricerche di Biologia della Selvaggina. 1993;21: 67–100.

[71] Leclerc M, Leblond M, Le Corre M, Dussault C, Côté SD. Determinants of migration trajectory and movement rate in a long-distance terrestrial mammal. J Mammal. 2021.

[72] Atwood EL. Life History Studies of Nutria, or Coypu, in Coastal Louisiana. J Wildl Manage. 1950;14: 249–265.

[73] Jang J-H, An J-H. Assessing future climate change impact on hydrologic and water quality components in Nakdong River Basin. J Korea Water Resour Assoc. 2012;45: 1121–1130 (in Korean with English abstract).

[74] Rousset F. Genetic differentiation and estimation of gene flow from *F*-statistics under isolation by distance. Genetics. 1997;145: 1219–1228. 9093870 10.1093/genetics/145.4.1219 PMC1207888

[75] McRae B, Beier P. Circuit theory predicts gene flow in plant and animal populations. Proc Natl Acad Sci USA. 2007;104: 19885. doi: 10.1073/pnas.0706568104 18056641 PMC2148392

[76] Hanks EM, Hooten M. Circuit theory and model-based inference for landscape connectivity. J Am Stat Assoc. 2013;108: 22–33.

[77] Aliev FF. Contribution to the study of nutria-migrations (*Myocastor coypus*). Saugetierkundliche Mitteilungen. 1968;16: 301–303.

[78] Dixo M, Metzger JP, Morgante JS, Zamudio KR. Habitat fragmentation reduces genetic diversity and connectivity among toad populations in the Brazilian Atlantic Coastal Forest. Biol Conserv. 2009;142: 1560–1569.

[79] Aliev FF. Dispersal of nutria in the USSR. J Mammal. 1965;46: 101–102.

